# Quality control questions on Amazon’s Mechanical Turk (MTurk): A randomized trial of impact on the USAUDIT, PHQ-9, and GAD-7

**DOI:** 10.3758/s13428-021-01665-8

**Published:** 2021-08-06

**Authors:** Jon Agley, Yunyu Xiao, Rachael Nolan, Lilian Golzarri-Arroyo

**Affiliations:** 1 Prevention Insights, Department of Applied Health Science, School of Public Health Bloomington, Indiana University Bloomington, 809 E. 9th St, Bloomington, IN 47405 USA; 2 School of Social Work, Indiana University Bloomington, Bloomington, IN USA; 3 School of Social Work, Indiana University-Purdue University Indianapolis (IUPUI), Bloomington, IN USA; 4 Department of Environmental & Public Health Sciences, College of Medicine, University of Cincinnati, Cincinnati, OH USA; 5 Biostatistics Consulting Center, School of Public Health Bloomington, Indiana University Bloomington, Bloomington, IN USA

**Keywords:** data quality, crowdsourced sampling, MTurk, reproducibility

## Abstract

Crowdsourced psychological and other biobehavioral research using platforms like Amazon’s Mechanical Turk (MTurk) is increasingly common – but has proliferated more rapidly than studies to establish data quality best practices. Thus, this study investigated whether outcome scores for three common screening tools would be significantly different among MTurk workers who were subject to different sets of quality control checks. We conducted a single-stage, randomized controlled trial with equal allocation to each of four study arms: Arm 1 (Control Arm), Arm 2 (Bot/VPN Check), Arm 3 (Truthfulness/Attention Check), and Arm 4 (Stringent Arm – All Checks). Data collection was completed in Qualtrics, to which participants were referred from MTurk. Subjects (*n* = 1100) were recruited on November 20–21, 2020. Eligible workers were required to claim U.S. residency, have a successful task completion rate > 95%, have completed a minimum of 100 tasks, and have completed a maximum of 10,000 tasks. Participants completed the US-Alcohol Use Disorders Identification Test (USAUDIT), the Patient Health Questionnaire (PHQ-9), and a screener for Generalized Anxiety Disorder (GAD-7). We found that differing quality control approaches significantly, meaningfully, and directionally affected outcome scores on each of the screening tools. Most notably, workers in Arm 1 (Control) reported higher scores than those in Arms 3 and 4 for all tools, and a higher score than workers in Arm 2 for the PHQ-9. These data suggest that the use, or lack thereof, of quality control questions in crowdsourced research may substantively affect findings, as might the types of quality control items.

**Supplementary Information:**

The online version contains supplementary material available at 10.3758/s13428-021-01665-8.

Over the last decade, there has been an increasing proliferation of psychological, bio-behavioral, public health, and other social research using online data collection platforms like Amazon’s Mechanical Turk (MTurk). In some fields, this surge has been notable; one recent report indicated a 2117% increase in management research studies using MTurk from 2012 to 2019 (Aguinis et al., 2020). Student-driven research, such as master’s theses and dissertations, has also begun pivoting to MTurk (Adesida, 2020; Brenner, 2020). This growth is reasonable, as experiments and other studies can benefit from the substantively larger pool of available participants on the Internet than traditional subject pools (Peterson, 2015). Further, it is possible that the COVID-19 pandemic may increase interest in online research platforms like MTurk, as faculty members work remotely more often (Flaherty, 2020). Thus, methods research identifying best practices for crowdsourced sampling is of high importance.

MTurk is not explicitly designed for research, though platforms like CloudResearch offer third-party MTurk research optimization tools for a fee (CloudResearch.com, 2021). The core MTurk program allows “requesters” to create a task and specify a payment amount for completion. Amazon collects a lump sum payment amount, along with a surcharge, then lists the task on a dashboard for all qualified “workers” to complete. Workers assert that they have completed a task, and requesters verify and approve the payment. Workers are anonymous to requesters, but not to Amazon (Amazon.com, 2020). For research, workers typically are asked to complete surveys and experiments embedded in an external platform (e.g., Qualtrics), with randomly generated completion IDs used for payment verification. As a result, conducting studies with MTurk requires a new set of procedures to be overlaid on standard protocols (e.g., steps to avoid fraudulent payment claims).

## Evolution of digital crowdsourced research

Early studies found MTurk to be effective for collecting surprisingly high-quality data for purposes of both clinical and non-clinical research (Buhrmester et al., 2011; Chandler & Shapiro, 2016; Johnson & Borden, 2012). As the number of studies using MTurk grew, additional research focused on best-practice approaches to what is now termed “crowdsourced sampling” (Keith et al., 2017; Kim & Hodgins, 2017). However, substantive threats to data quality began to emerge (Kim & Hodgins, 2020). These include, but are not limited to:

- inattentive workers, who can contribute statistical noise, though this issue has been identified in most online survey research (Berinsky et al., 2014);
- intentionally dishonest workers, or those who misrepresent things about themselves (Hydock, 2018);
- “bots,” which are programs that mimic workers (Buchanan & Scofield, 2018); and
- virtual private networks (VPNs), which allow workers to use a fake or concealed Internet protocol (IP) address. This can affect sampling approaches relying on Amazon’s embedded location identifier, as well as overall quality of the data, since workers using fake IP addresses often provide lower quality data (Dennis et al., 2019).

## Quality of crowdsourced samples from MTurk

To mitigate concerns about data quality, Amazon offers a “Master Worker” distinction, but using such workers is more expensive and did not appear to affect research data quality in a recent study (Loepp & Kelly, 2020). Other researchers have proposed a variety of caveats to maximize the utility of MTurk data, concluding that MTurk samples remain valid for many research purposes, but that researchers must especially consider: (a) mitigating risk of location/IP spoofing/identity via VPNs or bots (Dennis et al., 2019; Kennedy et al., 2020; Mellis & Bickel, 2020), (b) controlling for misrepresentation, especially for “rare” data points, such as “having managerial hiring experience” (MacInnis et al., 2020), and (c) managing inattentive responses (Barends & Vries, 2019; Mellis & Bickel, 2020).

Solutions to these concerns vary widely both in terms of recommendations and implementation.

- Identification of individuals using a VPN to mimic a US location has been demonstrated on the front-end by requesting workers to name an image that has regional nomenclature, such as an eggplant (Kennedy et al., 2020), and bots may be identified more broadly by restricting IP addresses (Mellis & Bickel, 2020). There is some evidence suggesting that bots or ‘nonhuman’ responses can be identified using post hoc statistical techniques, but with the underlying assumption that such systems respond at random (Dupuis et al., 2019). Other approaches, such as integrating third-party tools that contain databases of suspected VPN IP addresses, have also been studied (Kennedy et al., 2020).
- Misrepresentation or dishonest responding can be identified by proposing a fictitious relationship or identity characteristic (MacInnis et al., 2020). Importantly, it should be a characteristic that is impossible to possess (not just rare). It may also be possible to minimize this risk by not advertising eligibility criteria in cases where specific attributes are sought (e.g., hiring managers) (MacInnis et al., 2020).
- The most common approach to addressing inattentive respondents seems to be the use of “attention check” questions (Berinsky et al., 2014; Mellis & Bickel, 2020), though there is ongoing discussion about what constitutes an appropriate attention check (Prolific, 2018). In other cases, minimum reading speeds (time measurements) have been utilized, as has inspection for long strings, such as participants who answer “5” for many questions in a row (Ophir et al., 2019), though the latter case would not identify random patterns of response. There is also some evidence that noncompliant or inattentive responses can be identified *post hoc* using statistical approaches (Barends & Vries, 2019).

Despite these concerns, most articles proposing solutions emphasize that research using MTurk, and crowdsourced sampling more generally, remains valid and useful when conducted carefully. Studies have found that well-crafted studies using MTurk can produce data that is representative of multiple U.S. subpopulations, such as older adults and the U.S. labor force (Kraiger et al., 2020; Ogletree & Katz, 2020). Further, for certain characteristics like overall cognitive ability, or beliefs about privacy and security, MTurk samples have been found to be representative of national samples (Merz et al., 2020; Redmiles et al., 2019). MTurk has also been used successfully to replicate experimental psychology research for which results of the original, offline experiment are considered valid (Ganduillia et al., 2020). Nonetheless, crowdsourced sampling is different because of the explicitly transactional nature of the process (e.g., “workers” completing a “task” rather than “participants in a research study”). Thus, any unique risks to data quality introduced by this framework must be identified and managed.

## Our study

As described above, prior research largely has focused on the types of threats to validity. Few explicit recommendations exist for quality control procedures (e.g., assertions of gold-standard approaches), and little is known about how specific quality assurance approaches affect study outcomes. Therefore, given the literature indicating the need to manage VPNs/bots, misrepresentation, and inattention, we conducted a randomized, controlled experiment with four study arms (Control Arm, Bot/VPN Arm, Truth/Attention Arm, and Stringent Arm). The purpose of the study was to determine the absolute numeric difference, as well as differences in magnitude, skewness, and standard deviation, of different quality control procedures on outcomes from three self-administered digital tools used in a cross section of fields focused on mental health and substance use: the U.S. Alcohol Use Disorder Identification Test (USAUDIT) (Higgins-Biddle & Babor, 2018), the Patient Health Questionnaire (PHQ-9) (Kroenke et al., 2001), and the screener for Generalized Anxiety Disorder (GAD-7) (Spitzer et al., 2006).

These instruments (or similar, such as the AUDIT-C) have been used in recent crowdsourced studies on MTurk to explore a variety of important topics, such as associations between loneliness, depression, and COVID-19 (Killgore et al., 2020), relationships between sleep debt and anxiety (Dickinson et al., 2018), temporal relationships between day-level cravings and alcohol use (Jain et al., 2021), and the efficacy of Internet interventions for unhealthy alcohol use (Cunningham et al., 2019). At the same time, clinical studies have noted differences in self-reported prevalence of depression and anxiety between adult MTurk samples and other data sources, such as adult community samples or undergraduate research samples. Often, but not always, anxiety and depression have appeared to be more prevalent in samples from MTurk, and researchers have encouraged exploration of why this might be the case (Arditte et al., 2016; van Stolk-Cooke et al., 2018; Ophir et al., 2019; Engle et al., 2020). In response to this need, our methodological research provides the rapidly growing number of scholars using crowdsourced sampling with objective data indicating the expected impact and utility of multiple different quality-control procedures on crowdsourced data assessing depression, anxiety, and risky alcohol use.

We proposed two exploratory, preregistered hypotheses (Agley et al., 2020).

1. *Outcome scores from each of the three screening tools would be significantly different for each pairwise comparison of study arms, except for the pairing (Bot/VPN with Truth/Attention)*. We expected that each additional form of quality control would affect outcomes on all screening tools, except that we were agnostic as to whether there would be a meaningful difference between the different types of quality control (e.g., that the Bot/VPN control and the Truth/Attention control would produce differential effects). Thus, our hypotheses were based on the stringency of control mechanics by frequency count (e.g., 0, 1, 1, or 2 approaches within the arm), and we expected differences between each pair except the 1:1 pairing.
2. *Standard deviations for outcome scores from each of the three screening tools would be significantly different for each pairwise comparison of study arms, except for the pairing (Bot/VPN with Truth/Attention).* We expected that each additional form of quality control would affect outcome score distribution around the mean by reducing the frequency of random responses. As above, we did not expect a pairwise difference between the Bot/VPN and Truth/Attention arms.

## Method

### Preregistration

Key aspects of this study, including measures, hypotheses, and study design, were preregistered using the Open Science Framework (OSF) Registration platform (Agley et al., 2020).

### Participants

#### Sample size

We recruited 1100 participants (with replacement in some arms, see *Design*). Our *a priori* power analysis indicated that using a fixed effects ANOVA to detect an overall difference in means between four study arms, this sample would allow detection of a difference with effect size *f* = 0.10 (*F* = 2.61) at power 0.80, two-tailed alpha 0.05. With equal allocation, each arm was planned to have 275 subjects.

#### Recruitment

Subjects were recruited on November 20–21, 2020, using Amazon’s MTurk platform. We used MTurk specifications similar to those that we successfully used in our own prior research (Agley & Xiao, 2020). Eligible workers were required to: claim U.S. residency, have a successful task completion rate > 95%, have completed a minimum of 100 tasks, and have completed a maximum of 10,000 tasks. In addition, workers must be age 18 or older to join MTurk, setting a default minimum age for the study.

#### Compensation

Participants were paid $1.10 USD upon successful completion of the study but were not paid if they failed quality control checks. The informed consent statement warned participants: “This survey may include checks to screen out bots and individuals who are not eligible to take the survey. If you are screened out in this manner, the survey will end, and you should return the HIT in order to avoid your work being rejected.” In addition, the checks (see Table 1) all were in the first section of the study to avoid uncompensated data collection.

**Table 1:** Quality control (intervention) information

Arm name	Quality control questions	Rationale
Arm 1: Control/No Treatment	No additional exclusion criteria were appended to the basic eligibility requirements.	Control Arm.
Arm 2: Bot/VPN Check	(a) “If you had an emergency, what telephone number would you dial?” with the response options [112, 911, 000, and 119], each of which is a real emergency number in a different area of the world. (b) Participants were shown an image of an eggplant and asked, “What is the name of this vegetable?” with the response options [guinea squash, brinjal, aubergine, and eggplant], which are the four most common names of the vegetable.	(a) Since this was a U.S.-based sample, and respondents were at least age 18, it was expected that true U.S.-based participants would select 911. However, workers using a VPN to mimic a US-based IP address were hypothesized to select their own regional numbers, if present. Our experience in prior studies indicated that a meaningful number of supposedly U.S.-based workers would fail to select 911 (Agley & Xiao, 2020). (b) It was suspected that all but highly sophisticated bots would fail to directly identify an eggplant by name given only an image. Further, this functioned as a secondary VPN-check because the four names provided as response options are regional, with eggplant being standard terminology in the U.S.
Arm 3: Truthfulness/Attention Check	(a) “In the past 2 years, have you ever traveled to, or done any business with entities in, Latveria?” with response options [no, never; yes, but not within the past 2 years; yes, I have done so within the past 2 years]. (b) “Research has suggested that a person’s favorite color can tell us a lot about the way that they think about other people. In this case, however, we would like you to ignore this question entirely. Instead, please choose all of the response options provided. In other words, regardless of your actual favorite color, click all of the answers.” Respondents were provided with responses [red, blue, yellow, green, purple] but needed to select all five to demonstrate careful reading of the prompts. (c) “When you were in school, how hard did you work on your studies? In answering this question, please ignore everything else and select the final option indicating that you don’t really remember.” Responses were [I worked incredibly hard in school, I worked moderately hard in school, I didn’t work very hard in school, and I don’t recall how hard I worked]. Selecting anything but the last option indicated inattention.	(a) Latveria is a fictional nation ruled by Doctor Doom in the Marvel Comic Universe. This was an assessment of truthful response, with particular emphasis on the increased risk for “rare” datapoints (MacInnis et al., 2020). (b and c) In addition to the literature cited within the manuscript, our own experience also suggested that a meaningful segment of workers would be inattentive (Agley & Xiao, 2020).
Arm 4: Stringent Check	All questions from Arm 2 and Arm 3 were included in this arm.	This arm assessed whether there was a differential outcome when the approaches from Arm 2 and Arm 3 were combined.

#### Ethics approval and consent to participate

This study was reviewed by the Indiana University Institutional Review Board (IRB) prior to being conducted (#2011696997). All participants digitally indicated consent but were not informed that they would be randomized to different arms, nor that the purpose of the study was to assess the effects of data quality-control techniques. The only statement describing the study content within the SIS was, “This study will ask a series of questions about your mood, whether you have felt anxious recently, and your alcohol consumption.” A waiver for this incomplete informed consent was approved as part of the IRB review.

### Measures

Three screening tools were completed in each of the four arms: the USAUDIT (ten questions), PHQ-9 (nine questions), and GAD-7 (seven questions) (Higgins-Biddle & Babor, 2018; Kroenke et al., 2001; Spitzer et al., 2006). In those and numerous other studies, each screening tool has been validated and found reliable for self-administration in adult populations; unique scoring instructions are described in the cited studies as well, so we computed summed continuous variables for each instrument according to the established rules, with allowable ranges for USAUDIT [0 to 46], PHQ-9 [0 to 27], and GAD-7 [0 to 21].

Participants’ self-reported sociodemographic characteristics were collected for gender, ethnicity, race, age, and highest completed level of education. Question wording for each metric is available on OSF (Agley et al., 2020).

### Procedures

This was a single-stage, randomized controlled trial with equal allocation to each study arm (1:1:1:1 allocation). The size of each arm was fixed at the point of sampling but varied slightly for analysis (see Data cleaning). The intervention was a set of exclusion criteria for data collection procedures that varied by study arm (see Table 1 for details and rationale).

- Arm 1 was a control arm that contained no additional quality-control procedures beyond the standard eligibility requirements (see Participants).
- Arm 2 was a bot/VPN check that asked participants to select the telephone number they call in the event of an emergency and to correctly identify a drawn image of an eggplant.
- Arm 3 was a truthfulness/attention check that asked participants if they had done business with Latveria (a country that exists only in the Marvel Comic Universe) and then required them to pass two attention-check questions.
- Arm 4 was a stringent arm that combined the checks from Arms 2 and 3.

#### Allocation and storage

The allocation sequence was managed using the Randomizer tool in Qualtrics (Qualtrics, 2020). Allocation concealment was ensured because the procedure was automated and occurred after consent was processed. All data were collected and stored using the Qualtrics XM platform, which enables direct export to multiple formats (e.g., CSV, Excel, SPSS).

### Data cleaning

#### Core concepts

With MTurk, workers are not paid directly by researchers, but are instead provided with a unique random ID, which they enter into Amazon’s platform for verification. Thus, researchers must resolve discrepancies between the local list of IDs and the list submitted by workers for payment. In some cases, fraudulent ID submission may require a small number of additional surveys to be fielded, which was the case here.

**Fig. 1:**
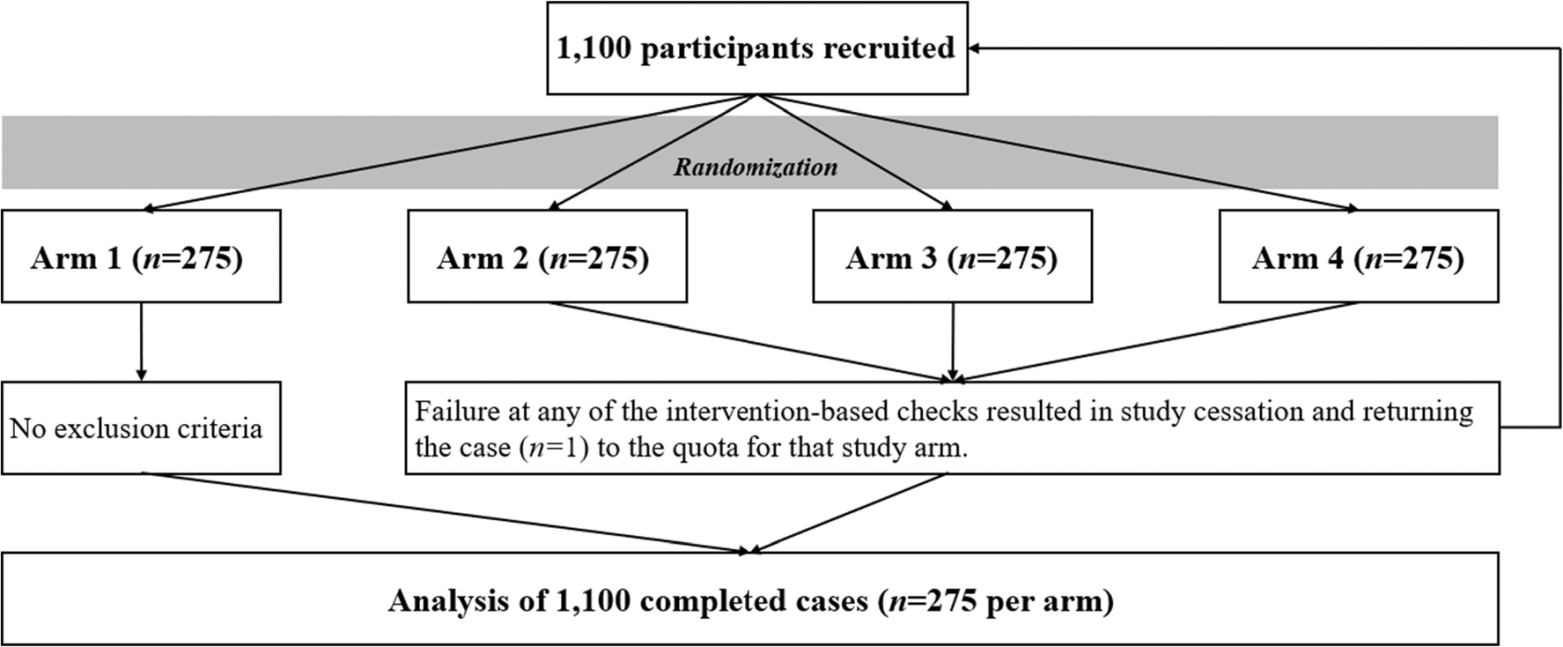
Conceptual CONSORT diagram

We paid for 1100 workers to complete our survey. In theory, the CONSORT flow diagram for this trial would look similar (Fig. 1). However, the separation between MTurk and Qualtrics (the survey platform) meant that there was an intermediary data-cleaning step that occurred while the study still was “in the field.” Specifically, several things could be true or false in each submitted case:

- Workers could (a) file for payment (submit their random ID generated by Qualtrics to MTurk for review) or (b) not.
- Workers could (a) submit their survey to Qualtrics, or (b) they could close the survey or Internet browser window before submitting it. Importantly, survey submission occurred at termination of the study, which occurred either when the questionnaire was fully completed or when the worker failed a quality control section. This meant that users could submit, or fail to submit, their responses in Qualtrics regardless of whether they were screened out by quality control measures or successfully finished the study. Thus, a “submitted survey” was different from an “unsubmitted survey,” and both were different from a “usable survey” where a participant successfully reached the questionnaire *and* submitted it.
- Workers could (a) submit a real ID provided by the study or could (b) submit a fake random ID, either by guessing based on IDs used by other studies, or by learning the ID pattern from an MTurk forum or website, though this occurs infrequently (Chandler et al., 2014).

#### Midstream assessment of the data

**Fig. 2:**
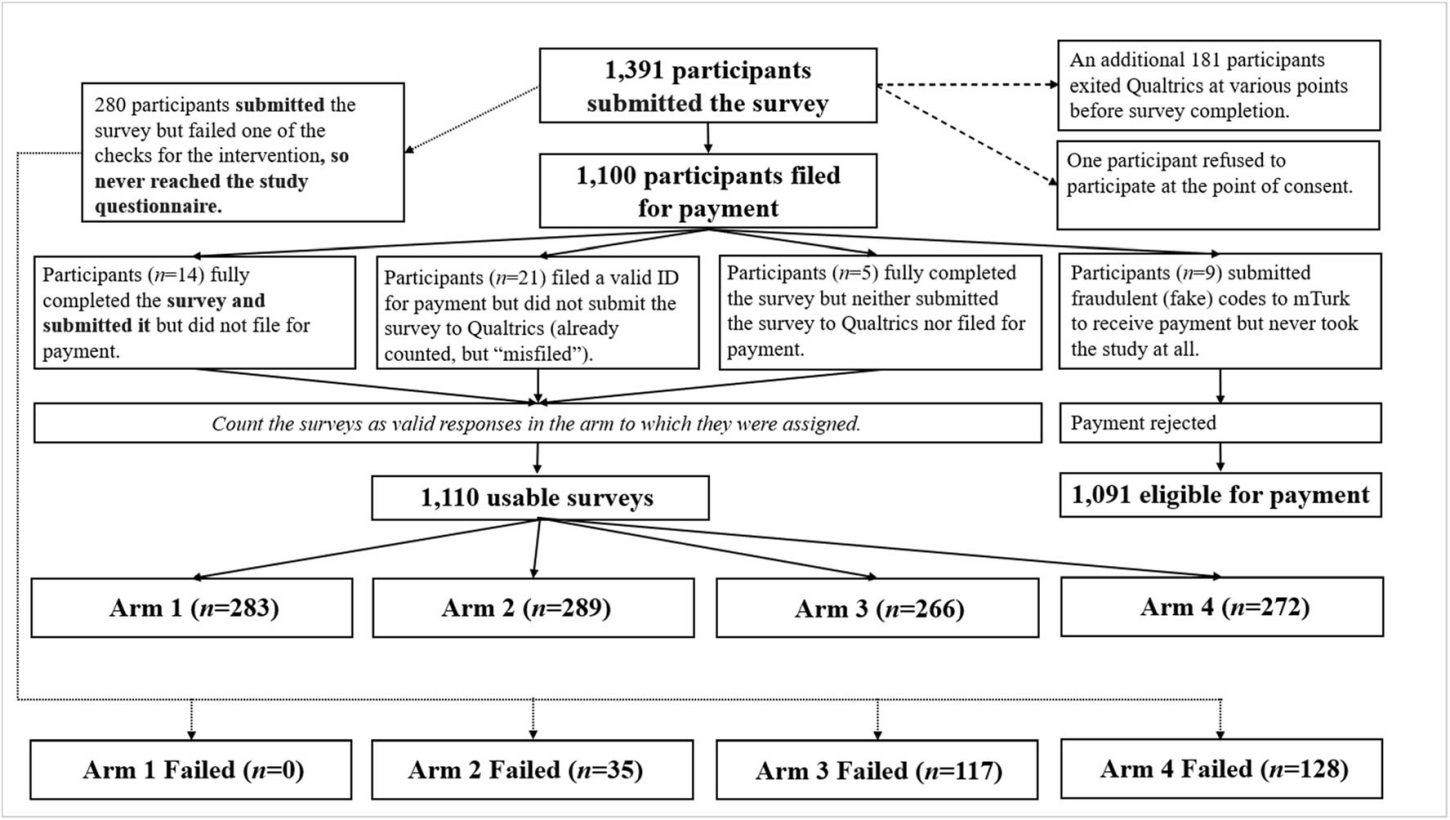
Interim data management

When 1100 workers had filed for payment, we had 1091 submissions eligible for payment, 1110 usable surveys, 1391 submitted surveys (including one refusal), and 181 unsubmitted surveys. We prepared a diagram to illustrate the computation of these numbers (see Fig. 2). At this point, we also cross-checked frequencies to validate the extant dataset (see the first portion of the analytic syntax in Attachment 1 and timestamped partial data in Attachment 2).

Ideally, randomization would occur after quality control checks, but the nature of the study, where the intervention *was* the quality control check, required randomization beforehand. Further, the rapid pace of response submission for a study on MTurk frequently meant that multiple people could be sorted into an arm when the quota was almost full, resulting in slight overage for that arm. This issue was compounded by payment claim discrepancies. Thus, as shown in Fig. 2, there was some variability in arm sizes. We opted not to alter the quotas or otherwise externally influence the random assignment. In making this decision, we considered that our primary hypothesis would be tested with ANOVA, which has been suggested to be fairly robust even to moderate deviations in statistical assumptions (Blanca et al., 2017).

#### Finalizing data collection

To reach our planned recruitment of 1100 paid subjects, we re-opened the survey for a brief period for nine more participants, with random assignment to Arms 3 and 4 (since Arms 1 and 2 were full). There were no anomalies at the payment claim review stage, meaning we obtained nine more usable surveys. Technically, those nine subjects had a different allocation chance (0:0:1:1), but sensitivity analyses (see supplemental files) that excluded those subjects did produce different study outcomes, so the data were retained. We also had 15 additional submitted surveys (who failed the quality check) and seven more unsubmitted surveys, bringing the total of submitted surveys to 1415 (+24), and the number of unsubmitted surveys to 188 (+7).

#### Incorporating unsubmitted surveys

**Fig. 3:**
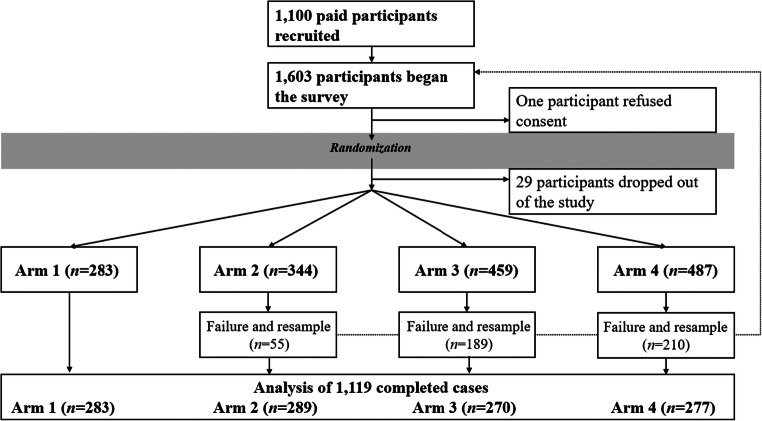
Actual CONSORT diagram

Unsubmitted surveys were merged with the dataset in the arm to which they were assigned using a binary decision heuristic. First, unsubmitted surveys for which the last answer provided before exiting was for a quality control question were considered to have been rejected from that arm. Second, unsubmitted surveys for which the last answer provided before exiting was *not* for a quality control question were considered to represent a participant who dropped out of the study (e.g., partial completion or non-response) for a reason unrelated to failing a quality control question. Thus, this dataset included 29 non-respondents (of whom 19 dropped out before the questionnaire, and ten dropped out during the USAUDIT; none dropped out during the PHQ-9 or GAD-7). We also assigned 20 additional rejections for Arm 2, 65 additional rejections for Arm 3, and 74 additional rejections for Arm 4. The final distribution of data is shown in Fig. 3, and the final analytic sample was 1119 usable surveys.

### Analyses

Data were analyzed, separately by screening tool, using analysis of variance (ANOVA), with study arm set as the independent variable and outcome score set as the dependent variable. Post hoc bivariate comparisons between study arms used Tukey’s HSD. In addition, reviewers requested exploratory analyses not present in the protocol. First, bivariate correlations between each screening tool were computed separately by study arm using Pearson correlation coefficients to verify that established correlations between these tools remained present in this study. Second, differences in sociodemographic variables were assessed across study arms using either Fisher’s exact tests with Monte Carlo estimation, or ANOVA, dependent on variable type.

Graphing software was utilized to generate visual distribution plots of the scores for each group to inspect differences in dispersion metrics (standard deviation, skewness, and kurtosis). Levene’s test of equality of variances based on medians (Nordstokke & Zumbo, 2007) was used to determine whether there was evidence of significant heterogeneity of variance between arms. To reduce missingness, respondents were required to answer each item on each page of the survey before proceeding. Thus, although we planned to analyze missing data using multiple imputation, the study structure was set up in such a way that almost no missingness was present.

### Results

A total of 1603 workers registered for the survey task on MTurk. Of those, one refused consent and 29 dropped out of the study. An additional 55 workers were rejected from Arm 2, 189 were rejected from Arm 3, and 210 were rejected from Arm 4, yielding the analytic sample of 1119 (which included 19 usable surveys where the worker did not submit to MTurk for payment; see Fig. 3). Sociodemographic characteristics of that sample are provided in Table 2. Samples in each arm tended to be slightly more male than female (54.4–57.8% male), except in Arm 3 (51.1% female). Participants were between 11.1% (Arm 3) and 20.1% (Arm 2) Hispanic/Latino, predominantly White (between 71.1% in Arm 4 and 79.2% in Arm 2), and each arm had generally normal distributions of education centered on bachelor’s degree. The mean age of respondents was narrowly bound across study arms, from 38.4 years to 39.9 years. These characteristics were relatively uniform between study arms for race, age, and education level. However, some significant differences were observed for ethnicity (*p* = .008) and gender (*p* = .025). Self-identified Hispanics were somewhat underrepresented in Arms 3 and 4. Further, small differences were observed in self-reported transgender and other gender-identity among arms, and self-identified females appeared more prevalent in Arm 3. Screening scores and analytic results are described subsequently, and are provided in Tables 3 and 4, respectively.

**Table 2:** Sociodemographic characteristics by study arm

	Arm 1		Arm 2		Arm 3		Arm 4		*p* value
	*n*	%	*n*	%	*n*	%	*n*	%	
**Gender**									.025^1^
Male	154	54.4	167	57.8	130	48.1	157	56.7	
Female	129	45.6	119	41.2	138	51.1	118	42.6	
Transgender	0	0.0	0	0.0	2	0.7	2	0.7	
Other	0	0.0	3	1.0	0	0.0	0	0.0	
**Ethnicity**									.008^1^
Hispanic/Latino	55	19.4	58	20.1	30	11.1	39	14.1	
Non-Hispanic/Latino	228	80.6	231	79.9	240	88.9	238	85.9	
**Race**									.570^1^
White	215	76.0	229	79.2	212	78.5	197	71.1	
Black/African American	38	13.4	35	12.1	28	10.4	44	15.9	
American Indian or Alaska Native	3	1.1	3	1.0	1	0.4	2	0.7	
Asian	22	7.8	12	4.2	20	7.4	20	7.2	
Native Hawaiian or Pacific Islander	0	0.0	1	0.3	1	0.4	1	0.4	
Other	2	0.7	3	1.0	4	1.5	5	1.8	
More than One Race	3	1.1	6	2.1	4	1.5	8	2.9	
**Education**									.747^1^
Less than High School	2	0.7	1	0.3	1	0.4	1	0.4	
High School Graduate / GED	51	18.0	56	19.4	48	17.8	63	22.7	
Associate's Degree	27	9.5	38	13.1	25	9.3	34	12.3	
Bachelor's Degree	146	51.6	136	47.1	127	47.0	120	43.3	
Master's Degree	51	18.0	51	17.6	62	23.0	51	18.4	
Doctoral or Professional Degree	6	2.1	7	2.4	7	2.6	8	2.9	
	***m***	***SD***	***m***	***SD***	***m***	***SD***	***m***	***SD***	***p*** **value**
**Age** (years)	38.4	*12.1*	39.3	*12.2*	39.9	*13.1*	38.9	*12.7*	.516^2^

1. Fisher’s exact test

2. ANOVA

**Table 3:** Screening scores by study arm

	Arm 1		Arm 2		Arm 3		Arm 4	
	*m*	*SD*	*m*	*SD*	*m*	*SD*	*m*	*SD*
USAUDIT (0-46)	13.6^1^	*10.2*	12.1^1^	*9.9*	9.1^1^	*8.4*	9.3^1^	*8.1*
PHQ-9 (0-27)	10.2^2^	*7.8*	8.5^3^	*7.2*	7.2^3^	*6.6*	7.1^3^	*6.8*
GAD-7 (0-21)	8.6^4^	*6.2*	7.3^4^	*5.9*	6.7^4^	*5.7*	6.2^4^	*5.6*

1. USAUDIT score indicates Zone 2

2. PHQ-9 score indicates Moderate Depression

3. PHQ-9 score indicates Mild Depression

4. GAD-7 score indicates Mild Anxiety

**Table 4:** ANOVA and Tukey HSD post hoc test scores

**USAUDIT**	**SS**	***Df***	**MS**	***F***	***p***
Between	4011.45	3	1337.15	15.78	< .001
Within	94480.65	1115	84.74	-	-
	**Mean Diff**	**SE**	**95%LL**	**95%UL**	***p***
Arm 1 vs. 2	1.57	0.77	– 0.41	3.55	.176
Arm 1 vs. 3	4.51	0.78	2.49	6.52	<.001
Arm 1 vs. 4	4.31	0.78	2.31	6.31	<.001
Arm 2 vs. 3	2.94	0.78	0.93	4.94	.001
Arm 2 vs. 4	2.74	0.77	0.75	4.74	.002
Arm 3 vs. 4	– 0.20	0.79	– 2.22	1.83	.995
**PHQ-9**	**SS**	***Df***	**MS**	***F***	***p***
Between	1807.76	3	602.59	11.94	<.001
Within	56290.00	1115	50.48	-	-
	**Mean Diff**	**SE**	**95%LL**	**95%UL**	***p***
Arm 1 vs. 2	1.69	0.59	0.16	3.22	.023
Arm 1 vs. 3	3.04	0.60	1.49	4.60	<.001
Arm 1 vs. 4	3.14	0.60	1.59	4.68	<.001
Arm 2 vs. 3	1.35	0.60	– 0.20	2.90	.112
Arm 2 vs. 4	1.44	0.60	– 0.10	2.98	.075
Arm 3 vs. 4	0.09	0.61	– 1.47	1.65	.999
**GAD-7**	**SS**	***Df***	**MS**	***F***	***p***
Between	859.48	3	286.49	8.35	<.001
Within	38267.90	1115	34.32	-	-
	**Mean Diff**	**SE**	**95%LL**	**95%UL**	***p***
Arm 1 vs. 2	1.23	0.49	– 0.03	2.49	.058
Arm 1 vs. 3	1.89	0.50	0.61	3.17	.001
Arm 1 vs. 4	2.32	0.50	1.05	3.59	<.001
Arm 2 vs. 3	0.66	0.50	– 0.62	1.93	.545
Arm 2 vs. 4	1.09	0.49	– 0.18	2.35	.122
Arm 3 vs. 4	0.43	0.50	– 0.86	1.72	.828

*SS = Sum of squares; Df = degrees of freedom; MS = mean squares; 95%LL/UL = 95% confidence interval of the mean difference, lower and upper levels; SE = standard error

### USAUDIT

The USAUDIT displayed good-to-excellent scale reliability, ranging from α = .884 in Arm 4 to α = .910 in Arm 1. Scores generally decreased from Arm 1 to Arms 3 and 4. Participants in Arm 1 reported a mean score of 13.64 (SD = 10.20), those in Arm 2 reported a mean score of 12.08 (SD = 9.88), those in Arm 3 reported a mean score of 9.14 (SD = 8.39), and participants in Arm 4 reported a mean of 9.33 (SD = 8.10). There was a significant overall difference in USAUDIT scores between the four study arms (*p* < .001). Post hoc testing using Tukey’s HSD identified that Arm 1 had a higher USAUDIT mean than Arm 3 (+ 4.51, 95% CI + 2.49 to + 6.52) and Arm 4 (+ 4.31, 95% CI + 2.31 to + 6.31), and that Arm 2 had a higher USAUDIT mean than Arm 3 (+ 2.94, 95% CI + 0.93 to + 4.94), and Arm 4 (+ 2.74, 95% CI + 0.75 to + 4.74).

### GAD-7

The GAD-7 displayed excellent scale reliability, ranging from α = .926 in Arm 3 to α = .933 in Arms 1 and 4. Scores decreased with each subsequent arm. Participants in Arm 1 reported a mean score of 8.56 (SD = 6.17), those in Arm 2 reported a mean score of 7.33 (SD = 5.87), those in Arm 3 reported a mean score of 6.67 (SD = 5.74), and participants in Arm 4 reported a mean of 6.24 (SD = 5.63). There was a significant overall difference in GAD-7 scores between the four study arms (*p* < .001). Post hoc tests found that the mean GAD-7 score in Arm 1 was higher than the score in Arm 3 (+ 1.89, 95% CI + 0.61 to + 3.17) and Arm 4 (+ 2.32, 95% CI +1.05 to + 3.59).

### PHQ-9

The PHQ-9 also displayed excellent scale reliability, ranging from α = .919 in Arm 3 to α = .938 in Arm 1. As with the GAD-7, scores decreased with each subsequent arm. Respondents in Arm 1 reported a mean score of 10.24 (SD = 7.77), those in Arm 2 reported a mean of 8.54 (SD = 7.18), respondents in Arm 3 reported a mean score of 7.19 (SD = 6.60), and those in Arm 4 reported a mean of 7.10 (SD = 6.78). There was a significant overall difference in PHQ-9 scores between the four study arms (*p* < .001). Post hoc testing identified a significantly higher mean PHQ-9 score for Arm 1 than Arm 2 (+ 1.69, 95% CI + 0.16 to + 3.22), Arm 3 (+ 3.04, 95% CI + 1.49 to + 4.60), and Arm 4 (+ 3.14, 95% CI + 1.59 to + 4.68).

### Differences in dispersion

**Fig. 4:**
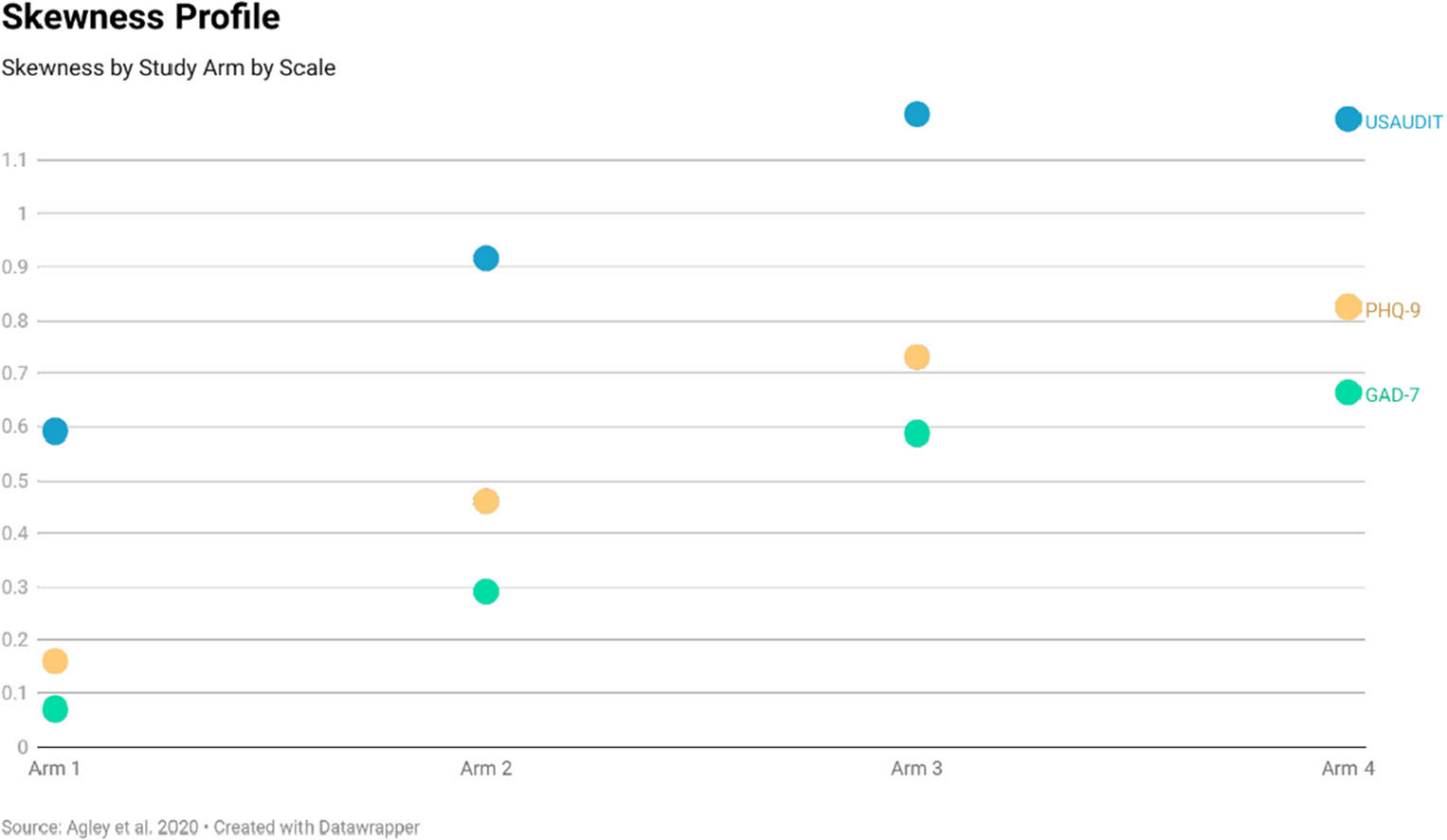
Skewness by Study Arm by Scale

**Fig. 5:**
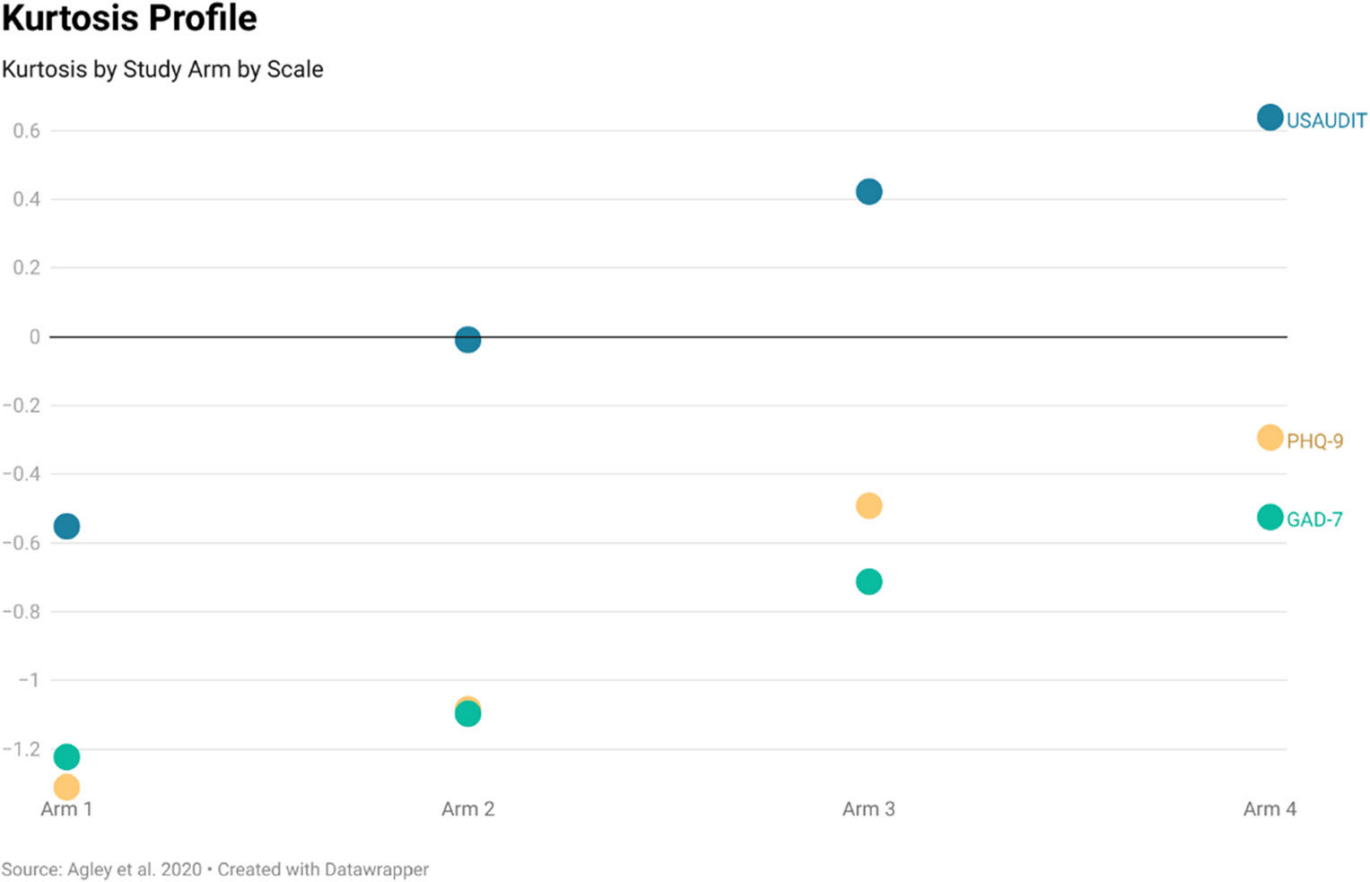
Kurtosis by study arm by scale

Levene’s tests based on the median (Nordstokke & Zumbo, 2007) clearly indicated heterogeneous dispersion across study arms for the USAUDIT (*F* = 10.685, *p* < .001) and PHQ-9 (*F* = 8.525, *p* < .001), and suggested heterogeneous dispersion for the GAD-7 (*F* = 2.681, *p* = .046). Although we originally proposed comparisons of standard deviation between arms, our prespecified visual inspection identified SD as a less useful metric than skewness and kurtosis in understanding these data (nonetheless, SD data are available through the supplemental files). Most notably, positive skewness was more evident in Arms 3 and 4 than in Arm 1 for each screening tool, with Arm 2 situated as a linear midpoint in skewness between Arm 1 and Arm 3. Visuals of skewness and kurtosis are available in Figs. 4 and 5.

### Correlations between screening tools

Table 5 contains bivariate correlation coefficients for each pair of screening tools separated by study arm. All analyses for each bivariate pair were statistically significant for each study arm (*p* < .001), as expected. However, there was some heterogeneity by arm in correlation coefficients, which ranged from *r* = 0.536 (Arm 3) to *r* = 0.580 (Arm 2) for the USAUDIT/PHQ-9 comparisons, from *r* = 0.403 (Arm 3) to *r* = 0.524 (Arm 1) for the USAUDIT/GAD-7 analyses, and from *r* = 0.855 (Arm 3) to *r* = 0.918 (Arm 1) for PHQ-9/GAD-7 comparisons.

**Table 5:** Correlations between screening scores by study arm

	**PHQ-9 (*r*)**	**GAD-7 (*r*)**
**Arm 1**		
USAUDIT	0.562	0.524
PHQ-9	1	0.918
**Arm 2**		
USAUDIT	0.580	0.472
PHQ-9	1	0.884
**Arm 3**		
USAUDIT	0.536	0.403
PHQ-9	1	0.855
**Arm 4**		
USAUDIT	0.543	0.477
PHQ-9	1	0.882

Note: All correlations significant at *p* < .001.

## Discussion

A substantial number of MTurk workers failed to pass basic data quality-control checks. As shown in Fig. 3, our approach to identifying users potentially using VPNs or bots to complete surveys generated fewer rejections (*n* = 55) than our questions to catch inattentive or dishonest respondents (*n* = 189), while the arm with both control types excluded even more (*n* = 210). Thus, our findings generally concurred with the literature, insofar as we identified similar types of noncompliant work to those that had previously been described. However, this study expands on prior research by providing causal evidence that selected quality control approaches appeared to have significant – *and directional* – effects on basic measures of alcohol use risk, generalized anxiety disorder, and depression.

## Impact of quality controls on screening scores

Our primary hypothesis regarding the impact of quality checks on the scores reported for three commonly used screening tools (USAUDIT, GAD-7, and PHQ-9) was partially supported, but our secondary hypothesis about dispersion was determined, after visual inspection of the data, to have been focused on the incorrect metric (standard deviation, rather than skewness and kurtosis), which is always a possibility when conducting preliminary studies. At the same time, our findings provided additional nuance beyond our exploratory theory – in particular, Arm 1 (no quality control) consistently produced scores that were significantly higher than both Arm 3 (truth/attentiveness checks) and Arm 4 (stringent quality control) for all three screening tools. The mean difference between Arm 1 and Arm 4 exceeded 9.4% of the total possible difference in score for each screening tool (e.g., the USAUDIT is scored from 0–46, and the mean difference between Arms 1 and 4 was 4.31). Further, in one case, the magnitude of the difference was clinically meaningful; the difference of 3.14 between Arm 1 and Arm 4 for the PHQ-9 is nearly the full numeric width of most zones of depression severity (Kroenke et al., 2001). In addition, score distribution (variability) was heterogeneous between study arms for all three tools, but especially for the USAUDIT and the PHQ-9. While we originally hypothesized pairwise differences in standard deviation, our visual inspection revealed that the dispersion metrics of interest are likely skewness and kurtosis, both of which display associations with study arm.

Interpreting these findings is complex, however. Based on our experiment, we can state that implementing the truth/attentiveness and stringent quality control checks caused the mean score for each screening tool to significantly decrease. Further, in the absence of quality control measures, participants more often imputed scores of greater absolute magnitudes than would be expected. This became evident on inspection of the skewness and kurtosis profiles. For example, we expected scores for all three of the selected screening tools to be positively skewed based on the US population distributions for risky alcohol use, depression, and anxiety. For example, PHQ-9 scores in the US population from the 2009–2014 National Health and Nutrition Examination Survey (NHANES) were positively skewed for all respondents (age 18+ years), with skewness values ranging from 1.8 to 2.2 by age group (Tomitaka et al., 2018). In our study, Arm 1, without quality controls, had skewness of only 0.2. The lower mean screening scores observed with the introduction of quality control approaches in Arms 2 through 4 were accompanied by increases in positive skewness. This suggests that adding quality control questions caused the distribution of screening scores to represent the shape of the expected population distribution more closely, though only to a point (Arm 4 had the highest positive skewness for PHQ-9 scores, at 0.8). This is consistent with research indicating that levels of depression measured on MTurk may exceed the general adult population even after accounting for invalid responses (Ophir et al., 2019).

At the same time, although we can indicate a causal link between these differences and the quality control approaches, we cannot determine why they occurred. One potential explanation is that inattentive, dishonest, or otherwise problematic participants (e.g., bots) did not add random noise to data, but rather systematically biased the data upwards by responding at the higher ends of the Likert-type items. Another possible explanation is that individuals who are more likely to participate dishonestly or inattentively in MTurk surveys are also more likely to have higher levels of depression, anxiety, or risky alcohol use. Regardless of the root cause of this finding, our study suggests that research using MTurk collect USAUDIT, PHQ-9, GAD-7, or similar screening data should clearly document the types of quality control utilized and consider the biases that its presence or absence may have on the data.

### Other practical implications

Based on our findings, we suggest several other ideas for consideration. First, in our study, control questions to identify bots or VPN users identified far fewer problematic cases than those to identify inattentive or dishonest users, though we cannot know whether this is because our bot/VPN questions were less efficacious or whether there were actually fewer noncompliant workers using those approaches. Regardless, it is likely insufficient for MTurk studies to implement controls only for bots or VPN users given the high prevalence, and demonstrable implications, of inattentive or dishonest workers. Second, as outlined in Table 1, our quality control checks were all based on established approaches derived from the literature. However, study nonnaïveté among MTurk workers is a salient concern (Chandler et al., 2014) that logically extends to quality control questions, so it is likely important to vary the specific content within each genre of question over time (e.g., the question about Latveria might be altered to use another fictional nation or to propose a vacation instead of business interaction). Third, it is not likely prudent to assume that noncompliant data obtained from crowdsourcing is primarily randomly distributed, or randomly distributed within structured patterns (e.g., selecting all “1” or all “5”); this may have implications for *post hoc* statistical quality controls.

Finally, somewhat separately, given the complexities identified in this study regarding how to prepare a CONSORT-style flow diagram for maximum transparency, we also suggest that researchers should consider whether to develop a preferred uniform reporting structure for randomized trials using crowdsourced sampling. Prior extensions to CONSORT guidelines typically have used a Delphi process and consensus meeting (e.g., Grant et al., 2018) to do so, but the procedures shared herein may prove to be useful discussion points.

## Limitations

We selected quality control measures that addressed different threats to validity, and that were conceptually in line with best-practice recommendations from recent reviews (e.g., Bauer et al.’s work, which was published in preprint while this study was ongoing) (Bauer et al., 2020). However, there remain no “gold standard” practices for quality-control measures, so we cannot be certain that we did not exclude a potentially key approach. Similarly, as with all work, these findings are subject to potential omitted variable bias. In addition, these results may not generalize to other crowdsourcing platforms like Prolific**.**

Finally, there is recent evidence that study framing may influence questionnaire responses on MTurk (Angus et al., 2021). A similar concern might also be raised about willingness to provide data about mental health and substance use. However, since we randomized participants after they had read about the study and agreed to participate, we believe that such effects would be minimized by the study design. In other words, all participants encountered the same framing and were asked the same questions about mental health and alcohol use, so these concerns would be less likely to influence comparability of data between study arms.

## Conclusions

This study found evidence that questions to facilitate quality control are likely important to consider and incorporate when conducting research on MTurk. As with all research, replication of these findings will be an important next step. Then, additional research to understand the unique nuances of crowdsourced sampling and experimentation is warranted, especially since controlling data quality improperly, or not at all, may introduce not just random variability, but directional variability.

## Supplementary Information

ESM 1 (Data Set 1, SPSS - see syntax for use) (SAV 541 kb)

ESM 2 (Data Set 2, SPSS - see syntax for use) (SAV 1.31 mb)

ESM 3 (Data Set 3, SPSS - see syntax for use) (SAV 1.47 mb)

ESM 4 (Data Set 4, CSV - see SAS syntax for use) (CSV 308 kb)

ESM 5 (CONSORT Table) (XLSX 11.5 kb)

ESM 6 (a) Electronic supplemental material 6 (SPSS Syntax) (DOCX 13.4 kb)

ESM 7 (b) Electronic supplemental material 7 (SAS Syntax for Fisher) (DOCX 17.3 kb)

## Data Availability

This study was preregistered with the Open Science Framework (OSF); https://osf.io/sv9ea. All data are available as supplemental files alongside this article.

## Declarations

### Code availability

Analytic code is available as a supplemental file alongside this article.
