# Supplementary material for: Quality control questions on Amazon’s Mechanical Turk (MTurk): A randomized trial of impact on the USAUDIT, PHQ-9, and GAD-7
Source: Behav Res Methods. 2021 Aug 6;54(2):885–97. doi: 10.3758/s13428-021-01665-8 (PMC8344397; doi:10.3758/s13428-021-01665-8)
Supplement: Supplementary file 6 — (a) Electronic supplemental material 6 (SPSS Syntax) (DOCX 13.4 kb) [file 13428_2021_1665_MOESM6_ESM.docx]

libname user '[PATH HERE]';

***Note that this is to be run with the specified file (SET 4) only. SAS was used because our version of SPSS does not incorporate Fisher. Due to uploading requirements the file appears as a CSV instead of an SAV file;

**proc** **import** datafile = "[PATH HERE]\Data Quality Project SET 4 Fisher ONLY.csv"

out=user.mTurkFisher;

**run**;

**proc** **datasets** library=user;

modify mTurkFisher;

format arm **8.**;

format overallgender **8.**;

format overallethnicity **8.**;

format overallrace **8.**;

format overalleduc **8.**;

**run**;

***Display these separately so they can be run one at a time on a slower machine, though with the MC estimate it should be fine for most systems;

**proc** **freq** data=mturkfisher;

tables arm*overallgender; exact fisher/MC;

**run**;

**proc** **freq** data=mturkfisher;

tables arm*overallrace; exact fisher/MC;

**run**;

**proc** **freq** data=mturkfisher;

tables arm*overallethnicity; exact fisher/MC;

**run**;

**proc** **freq** data=mturkfisher;

tables arm*overalleduc; exact fisher/MC;

**run**;
