# Supplementary material for: Quality control questions on Amazon’s Mechanical Turk (MTurk): A randomized trial of impact on the USAUDIT, PHQ-9, and GAD-7
Source: Behav Res Methods. 2021 Aug 6;54(2):885–97. doi: 10.3758/s13428-021-01665-8 (PMC8344397; doi:10.3758/s13428-021-01665-8)
Supplement: Supplementary file 7 — (b) Electronic supplemental material 7 (SAS Syntax for Fisher) (DOCX 17.3 kb) [file 13428_2021_1665_MOESM7_ESM.docx]

* Encoding: UTF-8.

***Frequency test before importing truncated surveys (run on Dataset 1 ONLY)

Frequencies Variables=Consent.

Frequencies Variables=A1Gender PHQ_9.

Temporary.

Select if Rejected2=0.

Frequencies Variables=A2Gender.

Frequencies Variables=Rejected2.

Temporary.

Select if Rejected3=0.

Frequencies Variables=A3Gender.

Frequencies Variables=Rejected3.

Temporary.

Select if Rejected4=0.

Frequencies Variables=A4Gender.

Frequencies Variables=Rejected4.

***All values add up. Total n=1,391, minus one negative consent, for n=1,390.

***Arm1=283, Arm2=289 + 35 rejection, Arm3=266 + 117 rejection, Arm4=272 + 128 rejection.

***Sum of all + 1 (non-consent) is 1,391. Sum of PHQ_9 (last question) = 1,391 - (Total Rejected).

***Thus, we conclude integrity of this dataset at this point.

***Note that you can rerun the syntax above for Dataset 2 in order to obtain the numbers under "Re-Collection" in the manuscript.

*************Data SET 3**************

Compute ID=$CaseNum.

Compute DropOut=0.

Execute.

*Compute cases that were rejected from Arm 2 but quit instead of completing

*Note that the following syntax used manual inspection of ID to generate a secondary variable to cross-validate this approach.

*It is redundant with the extant syntax but can be used to verify the # additional rejections from Arm 2.

**If (ID=1465) or (ID=1492) or (ID=1501) or (ID=1526) or (ID=1531) or (ID=1590) RejectedX=1.

**If (ID=1600) or (ID=1490) or (ID=1503) or (ID=1507) or (ID=1518) or (ID=1523) or (ID=1532) RejectedX=1.

**If (ID=1541) or (ID=1543) or (ID=1565) or (ID=1580) or (ID=1586) or (ID=1597) or (ID=1530) RejectedX=1.

If Sysmis(A2Race) and (A2Phone ne 2) and (Progress ne 100) Rejected2=1.

If (A2Plant ne 4) and (Progress ne 100) Rejected2=1.

Execute.

*Compute cases that were rejected from Arm 3 but quit instead of completing

If Sysmis(A3Ethnicity) and (Sum(A3Color_1 to A3Color_5) ne 5) and (Progress ne 100) Rejected3=1.

If Sysmis(A3Age) and (A3Latveria ne 1) and (Progress ne 100) Rejected3=1.

If (A3School ne 4) and (Progress ne 100) Rejected3=1.

Execute.

*Compute cases that were rejected from Arm 4 but quit instead of completing

If Sysmis(A4Ethnicity) and (Sum(A4Color_1 to A4Color_5) ne 5) and (Progress ne 100) Rejected4=1.

If Sysmis(A4Race) and (A4Phone ne 2) and (Progress ne 100) Rejected4=1.

If Sysmis(A4Plant) and (A4Latveria ne 1) and (Progress ne 100) Rejected4=1.

If Sysmis(A4Age) and (A4Plant ne 4) and (Progress ne 100) Rejected4=1.

If (A4School ne 4) and (Progress ne 100) Rejected4=1.

Execute.

*Compute true drop out cases.

If (Progress=48.00) DropOut=1.

If (Progress=74.00) DropOut=1.

If (Progress=80.00) DropOut=1.

If (Progress=88.00) DropOut=1.

If (Progress=92.00) DropOut=1.

Execute.

*Two cases passed an attention check then quit in Arm 3, one did so in Arm 4.

*Two at progress 55 quit after SIS but there was not a blanket rule for Progress=55, so added here. One of the same at 65.

If (ID=1482) or (ID=1462) or (ID=1559) or (ID=1499) or (ID=1519) or (ID=1502) DropOut=1.

Execute.

*Verify: Count of progress < 95 should now equal sum of Rejected 2 through 4 + DropOut.

Temporary.

Select If Progress<95.

Frequencies Variables = Progress Rejected2 Rejected3 Rejected4 DropOut.

***Compute final data for Arms.

Temporary.

Select If DropOut=0.

Frequencies Variables=A1Gender A2Gender Rejected2 A3Gender Rejected3 A4Gender Rejected4.

Compute Arm=0.

If (A1Gender>0) and (DropOut=0) Arm=1.

If (A2Gender>0) and (DropOut=0) and (Rejected2=0) Arm=2.

If (A3Gender>0) and (DropOut=0) and (Rejected3=0) Arm=3.

If (A4Gender>0) and (DropOut=0) and (Rejected4=0) Arm=4.

Execute.

Frequencies Variables=Arm.

******************Computing USAUDIT Scores***********************

*Recode from the Qualtrics numbers to the scoring mechanism from literature

Recode AUDIT1 (8=6) (6=5) (5=4) (4=3) (3=2) (2=1) (1=0).

Recode AUDIT2 AUDIT3a AUDIT3b (6=5) (5=4) (4=3) (3=2) (2=1) (1=0) (7=6).

Recode AUDIT4 AUDIT5 AUDIT6 AUDIT7 (5=4) (4=3) (3=2) (2=1) (1=0).

Recode AUDIT9 AUDIT10 (3=4) (1=0).

Execute.

Compute USAUDIT=Sum(AUDIT1 to AUDIT10).

Execute.

**View output by arm.

Sort Cases by Arm.

Split File separate by Arm.

Temporary.

Select If Arm>0.

Descriptives Variables=USAUDIT

/Statistics=MEAN STDDEV MIN MAX KURTOSIS SKEWNESS.

Temporary.

Select If Arm>0.

Frequencies Variables=USAUDIT

/Histogram=Normal

/Order=Analysis.

Split File off.

Temporary.

Select If Arm>0.

Oneway USAUDIT by Arm

/Missing Analysis

/STATISTICS HOMOGENEITY

/Posthoc=Tukey Gh Alpha(0.05).

******************Computing PHQ-9 Scores***********************

*Recode from the Qualtrics numbers to the scoring mechanism from literature

Recode PHQ_1 to PHQ_9 (4=3) (3=2) (2=1) (1=0).

Execute.

Compute PHQSCORE=Sum(PHQ_1 to PHQ_9).

Execute.

**View output by arm.

Sort Cases by Arm.

Split File separate by Arm.

Temporary.

Select If Arm>0.

Descriptives Variables=PHQSCORE

/Statistics=MEAN STDDEV MIN MAX KURTOSIS SKEWNESS.

Temporary.

Select If Arm>0.

Frequencies Variables=PHQSCORE

/Histogram=Normal

/Order=Analysis.

Split File off.

Temporary.

Select If Arm>0.

Oneway PHQSCORE by Arm

/Missing Analysis

/STATISTICS HOMOGENEITY

/Posthoc=Tukey Gh Alpha(0.05).

******************Computing GAD-7 Scores***********************

*Recode from the Qualtrics numbers to the scoring mechanism from literature

Recode GAD_1 to GAD_7 (4=3) (3=2) (2=1) (1=0).

Execute.

Compute GADSCORE=Sum(GAD_1 to GAD_7).

Execute.

**View output by arm.

Sort Cases by Arm.

Split File separate by Arm.

Temporary.

Select If Arm>0.

Descriptives Variables=GADSCORE

/Statistics=MEAN STDDEV MIN MAX KURTOSIS SKEWNESS.

Temporary.

Select If Arm>0.

Frequencies Variables=GADSCORE

/Histogram=Normal

/Order=Analysis.

Split File off.

Temporary.

Select If Arm>0.

Oneway GADSCORE by Arm

/Missing Analysis

/STATISTICS HOMOGENEITY

/Posthoc=Tukey Gh Alpha(0.05).

**************Correlation Analysis Requested in Review***********************

Sort Cases by Arm.

Split File separate by Arm.

Temporary.

Select If Arm>0.

Correlations

/Variables=USAUDIT PHQSCORE GADSCORE

/Print=Twotail Nosig

/Missing=Pairwise.

Split File off.

**************Demographic Analysis Requested in Review***********************

**This subsection was used to generate Set 4 for Fisher's Exact Test. That dataset is provided, and to use it you need to run the SAS syntax that came with the article.

Select If Arm>0.

Recode A1Gender A2Gender A3Gender A4Gender A1Ethnicity A2Ethnicity A3Ethnicity A4Ethnicity A1Age A2Age A3Age A4Age A1Race A2Race A3Race A4Race A1Educ A2Educ A3Educ A4Educ (Sysmis=0).

Execute.

Compute OverallGender=(A1Gender + A2Gender + A3Gender + A4Gender).

Compute OverallEthnicity=(A1Ethnicity + A2Ethnicity + A3Ethnicity + A4Ethnicity).

Compute OverallAge=(A1Age + A2Age + A3Age + A4Age).

Compute OverallRace=(A1Race + A2Race + A3Race + A4Race).

Compute OverallEduc=(A1Educ + A2Educ + A3Educ + A4Educ).

Execute.

Value Labels OverallGender

'1' Male

'2' Female

'3' Transgender

'4' Other.

Execute.

Value Labels OverallEthnicity

'1' Hispanic

'2' Non-Hispanic.

Execute.

Value Labels OverallRace

'1' White

'2' Black

'3' AI-AN

'4' Asian

'5' NH-PI

'6' Other

'7' More Than One.

Execute.

Value Labels OverallEduc

'1' Less than High School

'2' High School

'3' Associate

'4' Bachelor

'5' Master

'6' Doctoral or Professional.

Execute.

***Sensitivity analysis for the 9 subjects with unequal allocation chance.

Temporary.

Select If Arm>0 and (randID ne "R9125008") and (randID ne "R2713642") and (randID ne "R9273878") and (randID ne "R5944345") and

(randID ne "R8361816") and (randID ne "R5124578") and (randID ne "R8782496") and (randID ne "R2503771") and (randID ne "R4407655").

Oneway USAUDIT by Arm

/Missing Analysis

/STATISTICS HOMOGENEITY

/Posthoc=Tukey Gh Alpha(0.05).

Temporary.

Select If Arm>0 and (randID ne "R9125008") and (randID ne "R2713642") and (randID ne "R9273878") and (randID ne "R5944345") and

(randID ne "R8361816") and (randID ne "R5124578") and (randID ne "R8782496") and (randID ne "R2503771") and (randID ne "R4407655").

Oneway PHQSCORE by Arm

/Missing Analysis

/STATISTICS HOMOGENEITY

/Posthoc=Tukey Gh Alpha(0.05).

Temporary.

Select If Arm>0 and (randID ne "R9125008") and (randID ne "R2713642") and (randID ne "R9273878") and (randID ne "R5944345") and

(randID ne "R8361816") and (randID ne "R5124578") and (randID ne "R8782496") and (randID ne "R2503771") and (randID ne "R4407655").

Oneway GADSCORE by Arm

/Missing Analysis

/STATISTICS HOMOGENEITY

/Posthoc=Tukey Gh Alpha(0.05).
